# Urocortin-1 Mediated Cardioprotection Involves XIAP and CD40-Ligand Recovery: Role of EPAC2 and ERK1/2

**DOI:** 10.1371/journal.pone.0147375

**Published:** 2016-02-03

**Authors:** Eva Calderón-Sánchez, Ignacio Díaz, Antonio Ordóñez, Tarik Smani

**Affiliations:** 1 Grupo de Fisiopatología Cardiovascular, Instituto de Biomedicina de Sevilla-IBiS, HUVR/Universidad de Sevilla/CSIC, Seville, Spain; 2 Departamento de Fisiología Médica y Biofísica, Universidad de Sevilla, Seville, Spain; Emory University, UNITED STATES

## Abstract

**Aims:**

Urocortin-1 (Ucn-1) is an endogenous peptide that protects heart from ischemia and reperfusion (I/R) injuries. Ucn-1 is known to prevent cardiac cell death, but its role in the transcription of specific genes related to survival signaling pathway has not been fully defined. The aim of this study was to investigate the molecular signaling implicated in the improvement of cardiac myocytes survival induced by Ucn-1.

**Methods and Results:**

Ucn-1 administration before ischemia and at the onset of reperfusion, in rat hearts perfused in Langendorff system, fully recovered heart contractility and other hemodynamic parameters. Ucn-1 enhanced cell viability and decreased lactate dehydrogenase (LDH) release in adult cardiac myocytes subjected to simulated I/R. Annexin V-FITC/PI staining indicated that Ucn-1 promoted cell survival and decreased cell necrosis through Epac2 (exchange protein directly activated by cAMP) and ERK1/2 (extracellular signal–regulated kinases 1/2) activation. We determined that Ucn-1 shifted cell death from necrosis to apoptosis and activated caspases 9 and 3/7. Furthermore, mini-array, RT-qPCR and protein analyses of apoptotic genes showed that Ucn-1 upregulated the expression of CD40lg, Xiap and BAD in cells undergoing I/R, involving Epac2 and ERK1/2 activation.

**Conclusions:**

Our data indicate that Ucn-1 efficiently protected hearts from I/R damage by increasing the cell survival and stimulated apoptotic genes, CD40lg, Xiap and BAD, overexpression through the activation of Epac2 and ERK1/2.

## Introduction

Despite the considerable advances that have been made in the field of myocardial protection, ischemic heart disease represents a major public health problem and the main cause of mortality in the industrialized world [1]. Percutaneous transluminal angioplasty, fibrinolysis and cardioplegic solutions are some of the strategies developed to preserve the myocardial viability from ischemia. All these procedures involve myocardial reperfusion/reoxygenation after an ischemic episode. However, the subsequent reperfusion also activates various injury responses leading to necrosis, apoptosis and general heart dysfunction [1, 2].

Special interest has been made toward the endogenous protection elicited by the heart as a potent approach to limit heart lesions from I/R insult. In the last two-decade, urocortin peptides (Ucn-1, Ucn-2, Ucn-3), which belongs to the corticotropin-releasing factor (CRF) family [3], have emerged as a potential therapeutic agonist that improves heart performances and protects heart from I/R injuries [4]. In the cardiovascular system, urocortin binding to its G protein–coupled receptor (CRF-R_2_) is known to enhance cAMP production [5], which is classically related to PKA activation. However, a guanine nucleotide exchange factor (GEF) also activated directly by cAMP, named exchange protein activated by cyclic AMP (Epac) emerged as a new player of several cAMP-regulated processes in heart such as heart inotropism [6], cardiac myocytes hypertrophy [7], and Ca^2+^ handling in cardiac myocytes [8]. Previously, we have described that Epac and ERK1/2 are involved in urocortin-induced positive inotropism in rat hearts [9]. However, Epac role in cardioprotection has been barely studied.

Different mechanisms are implicated in the cardioprotection afforded either by Ucn-1 or Ucn-2, involving the rapid activation of protective signaling pathways [10], calcium-independent phospholipase A_2_ (iPLA_2_) and protein kinase C epsilon (PKCε) [11], or ERK1/2 [12, 13], among others. Urocortin also regulated cell survival and apoptosis during I/R injury, through caspase 3 inhibition [10], STAT3 [14] or p38MAPK activation [15]. We have shown recently that Ucn-1 administration only at the beginning of the reperfusion preserved heart contractility by the improvement of intracellular Ca^2+^ handling, which included the recovery of cells excitability, the inhibition of diastolic Ca^2+^ increase and the regulation of Na^+^/Ca^2+^ exchanger [16].

Herein, we explored the molecular pathway involved in Ucn-1 evoked heart protection with special emphasis on Epac and ERK1/2 on their role in cardiac myocytes survival. We also examined the effect of Ucn-1 on cell death pathways and its regulation of apoptotic genes, CD40lg, Xiap and BAD.

## Materials and Methods

All the experiments with animals were performed in accordance with the recommendations of the Royal Decree 53/2013 in agreement to the Directive 2010/63/EU of the European Parliament and approved by the local Ethics Committee on human Research of the “Virgen del Rocio” University Hospital of Seville and the Animal Research Committee of the University of Seville.

### *Ex vivo* Langendorff-perfused rat heart

Adult male *Wistar* rats weighing 250–350 g were heparinized (4 IU/g i.p.) and anaesthetized by intraperitoneal administration of an overdose of sodium thiopental (200 mg/Kg). The hearts were quickly removed, mounted on the aortic cannula of the Langendorff perfusion system apparatus and perfused with an oxygenated Krebs- Henseleit buffer (en mM; 118 NaCl, 4.7 KCl, 1.25 CaCl_2_, 1.2 KH_2_PO_4_, 1.2 MgSO_4_, 25 NaHCO_3_, and 5 glucose) as described previously [9, 17]. Before each experimental protocol was initiated, the isolated hearts were set at a mean arterial pressure of 60–80 mmHg and were allowed to stabilize at 37°C for 40 to 60 minutes. Chart Powerlab software (ADInstruments) was used for continuous recording throughout the experiments of heart rate, left ventricular developed pressure (LVDP), and maximum positive and negative derivative of left ventricular pressure (±dP/dt). The heart contractility under different treatments was evaluated by the analysis of +dP/dt, which corresponds to % increase of +dP/dt normalized to basal value after the period of stabilization.

The standard protocol of ischemia/reperfusion in perfused hearts was followed as described previously [16]. Group 1 of I/R: After stabilization, rat hearts were exposed to global ischemia (without aorta perfusion) during 40 minutes and 1 hour of reperfusion with freshly oxygenated solution. Group 2 corresponds to the pharmacological preconditioning with Ucn-1 (10 nM). After stabilization, Ucn-1 was applied 20 minutes before ischemia and 30 minutes at the beginning of reperfusion.

### Isolation of ventricular myocytes

The hearts were removed and mounted on a Langendorff perfusion apparatus. Ventricular myocytes were isolated using collagenase type II (251 IU/mL; Worthington Biochemical, Lakewood, NJ, USA) as described previously [9]. Subsequently, isolated cells were filtered, centrifuged and suspended in Tyrode solution containing (en mM): 130 NaCl, 1 CaCl_2_, 0.5 MgCl_2_, 5.4 KCl, 22 glucose, 25 HEPES, 0.4 NaH_2_PO_4_, 5 NaHCO_3_; pH was adjusted to 7.4 with NaOH. Cardiac myocytes were plated in control solution containing 1.8 mM CaCl_2_ at 37°C, and were submitted to a protocol of I/R using a simulated ischemic solution (mM): 142 NaCl, 3.6 KCl, 1.2 MgCl_2_, 1.8 CaCl_2_, 20 Hepes, 20 Lactate-Na, 20 sucrose (pH 6.2). Cells were then placed during 30 minutes in an incubator of 1% O_2_ and 5% CO_2_. Afterward, cells were incubated in control solution in a 21% O_2_ and 5% CO_2_ incubator during 18 hours. All the experiments were performed on Ca^2+^-tolerant rod-shaped myocytes.

### Treatment protocols

Group 1, control: Untreated cardiac myocytes.
Group 2, I/R: After stabilization in control solution, cells were exposed to simulated ischemia solution during 30 minutes followed by 18 hours of reperfusion with freshly control solution.
Group 3, Ucn-1: Same as group 2, but Ucn-1 (10 nM) was applied 20 minutes before ischemia followed by reperfusion.
Group 4, 8CPT: preconditioning with 8CPT (10 μM), specific agonist of Epac: Same as group 3 but 8CPT was applied instead of Ucn-1.
Group 5 and 6: Cells treated with either ESI-05 (10 μM, Epac2 inhibitor) or PD 098059 (5 μM, ERK1/2 inhibitor) 10 minutes before the addition of Ucn-1 (10 nM) following the same protocol as in group 3.

### Apoptosis and necrosis assays

The level of lactate dehydrogenase (LDH) was detected in supernatant of cells subjected to simulated I/R protocol according to the manufacturer’s instructions (Promega). Trypan Blue staining method was also used for detecting cell death in cardiac myocytes undergoing treatment with I/R ± Ucn-1 (10 nM). Briefly, after each treatment cells were washed three times with PBS, then cells were bathed in 0.5% of trypan blue for 10 minutes. Based on the classical cell counting, we acquired 4 random snapshots per each condition using 40x Objective of an Olympus microscope. Maximum projection was recorded and analyzed with “Image J” software to count the different proportion of trypan positive and negative cells by an investigator blinded to the treatment conditions. This process was repeated in triplicate for each condition and the experiment was replicated on 3 separate occasions. 100% indicates total number of trypan stained and unstained cells in each condition.

Double staining with annexin V-FITC and propidium iodide (PI) was also performed to examine apoptosis and necrosis in cultured cardiac myocytes. During apoptosis, an early and ubiquitous event is the exposure of phosphatidylserine at the cell surface, which is detected with annexin V-FITC. PI is used to determine the population of cells that have lost membrane integrity, an indication of late apoptosis or necrosis. Briefly, cardiac myocytes were seeded in a 6-channel μ-Slide (μ-Slide VI 0,4) from IBID. After treatments cardiac myocytes were incubated with 100 μl of the binding buffer supplied in the kit (Cat. 4830-01-K. Trevigen) and 1 μl of annexin-FITC reagent, during 60 minutes at 25°C. Then PI was added and incubated for 15 minutes and later washed before mounting the slides. Images were taken with confocal microscope Leica TCS SP2 microscopy (Leica). 5 snapshots per condition were acquired using a HCX PI Apo CS 40x objective in z-stacks intervals, and maximum projection was recorded and analyzed with “Image J” software to count the different proportion of cells that were labeled only with annexin (early apoptosis), or with PI and/or annexin and PI (necrosis or late apoptosis).

### Caspase 9, 8 and 3/7 activity

To determine the caspase activity we used the Caspase-Glo assay kit system from Promega; TB323 for Caspase 3/7, TB332 for Caspase 8, and TB333 for Caspase 9. Protein extracts from cultured cardiac myocytes were quantified by Bradford (Sigma Aldrich) method and were diluted at 1.5 μg/μl of protein. 10 μl of protein extract and 10 μl of Caspase-Glo reagent were added in a 384 flat and white multi-well dish in quadruplicate replicates and incubated for 40 minutes at room temperature. Luminescent signal was detected in a Luminoskan ascent microplate luminometer (Thermo Scientific).

### Western Blotting

Protein samples were extracted from cultured and treated cardiac myocytes and 40 μg of protein were subjected to SDS-PAGE (10% acrylamide) and electrotransferred onto PVDF membranes. Membranes were probed overnight at 4°C with specific primary antibodies in TTBS with 1% of BSA. CD40lg antibody was from sigma. BAD, phosphoBAD, Xiap, phosphoERK1/2 and ERK1/2 primary antibodies were from Cell Signaling. Detection was performed with the enhanced chemiluminiscence reagent ECL-plus (Amersham Bioscience) in the ImageQuant LAS 4000 mini (GE Healthcare). For quantification, the images were analyzed with “Image J” software using alpha-tubulin (Sigma) or GAPDH (Genetex) as housekeeping loading control.

### Analysis of mRNA expression

Total RNA was extracted from cultured cardiac myocytes using the RNeasy kit (Qiagen) according to the manufacturer’s instructions. RNA purity and concentration were assessed by measuring absorption at 260 nm and 280 nm. 1 μg of RNA were retro-transcribed to cDNA with the RT quantitec kit (Qiagen). qRT-PCRs were performed with the use of a Prism 7900HT Sequence Detection System, Taqman primers and probes technologies (Applied Biosystems). Fold change in gene expression was calculated using the comparative cycle threshold CT (ΔΔCT) method.

For apoptosis PCR array, we used plates from SA Biosciences (Cat. PAHS-012). Total RNA from Ucn-1 treated and no-treated cardiac myocytes (as described in cell treatment protocols) submitted to I/R was extracted and reverse transcribed into cDNA with the use of an RT2 First Strand Kit (SA Biosciences). The templates were combined with an RT2 SYBR Green qPCR Master Mix (SA Biosciences), and then equal aliquots of this mixture (25 μl) were added to each well of the same PCR Array plate that contained the pre-dispensed gene-specific primer sets. The qRT-PCR quantification was performed as described above by ΔΔCT method.

### Statistical analysis

Data analysis was carried out using SigmaPlot software, version 11.0. A sample size calculation was performed prior the start of this study. Group data are presented as mean ± S.E.M. Single or paired Student's t test was used to determine the statistical significance of the obtained data. The significance between multiple groups was evaluated using ANOVA followed by Tukey multiple comparison post hoc tests for comparing different groups. Data marked by *, **, *** were considered significantly different at p<0.05, p<0.01 and p<0.001 respectively.

## Results

### Urocortin-1 improves the hemodynamic performances in isolated rat hearts submitted to ischemia and reperfusion protocol

**Fig 1:**
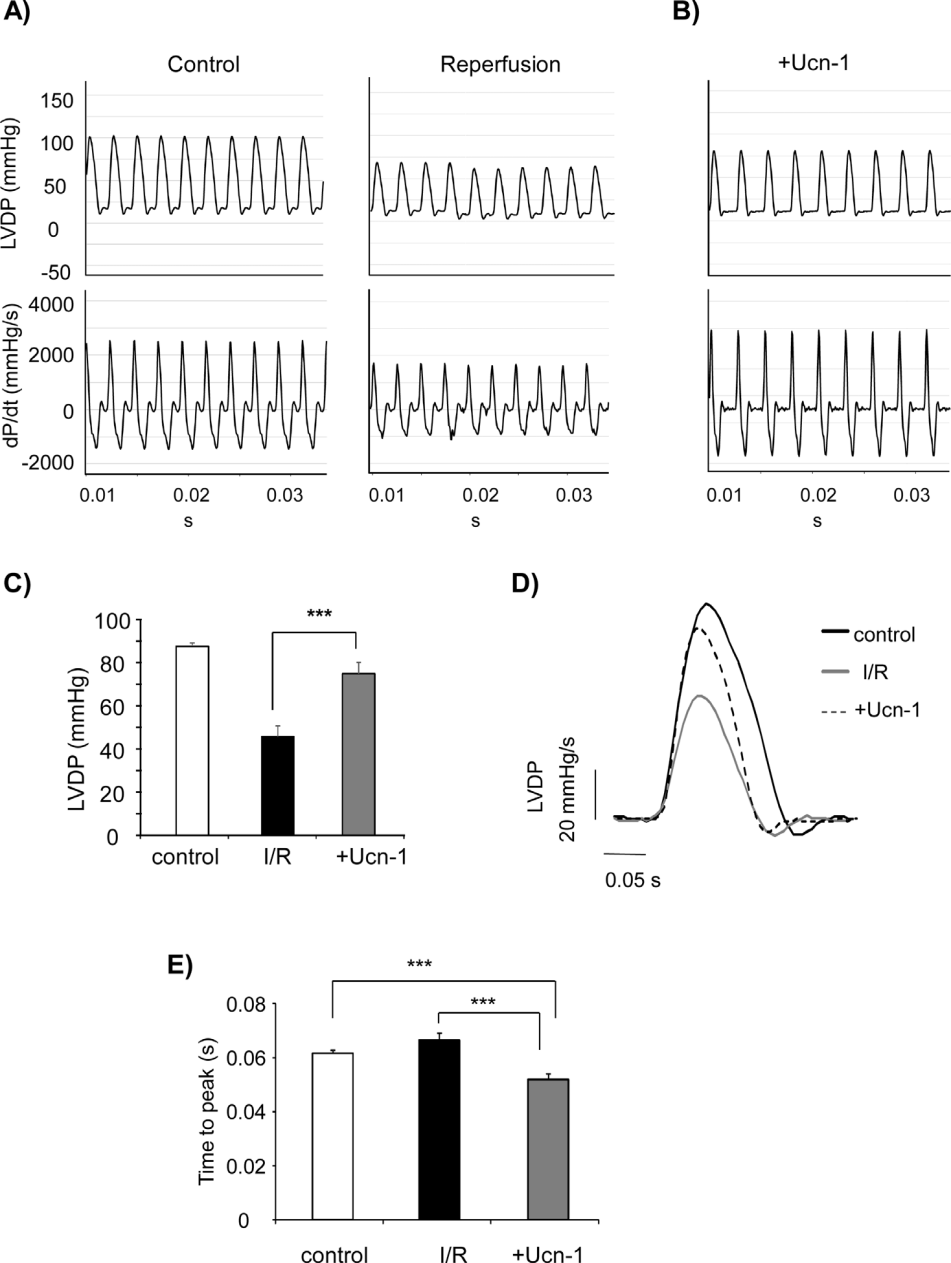
Effect of urocortin-1 on contractility in *ex vivo* isolated perfused rat hearts. **A**) Representative original recording of the left ventricular developed pressure (LVDP, upper tracing), and the derivative of left ventricular pressure (dP/dt, lower tracing) in Langendorff perfused heart before ischemia (control) and in reperfusion after 40 minutes of global ischemia (reperfusion). **B**) Representative recording in reperfusion of LVDP and dP/dt from heart treated 20 minutes with urocortin-1 (Ucn-1) before ischemia and 30 minutes at the onset of reperfusion. **C**) Graph shows summary data of LVDP expressed as the difference between maximum and basal values from control, ischemia and reperfusion (I/R), and Ucn-1 treated hearts. **D**) Superimposed representative single traces of LVDP from control, reperfusion after ischemia (I/R), and in reperfusion from Ucn-1-treated hearts. **E**) Summary data of time-to-peak of LVDP. Values are means ± s.e.m from 6 hearts. “***” indicates significance at p<0.001 of +Ucn-1 vs I/R.

The protective effects of Ucn-1 on heart contractility were analyzed in *ex vivo* Langendorff-perfused rat hearts submitted to I/R. Fig 1A–1C shows that hearts exposed to 40 minutes of ischemia and 60 minutes of reperfusion (I/R) suffered a significant decrease in the left ventricular developed pressure (LVDP) as well as in the positive and negative maximum derivative of left ventricular pressure (±dP/dt), indicating significant loss in heart function. The addition of Ucn-1 (10 nM) 20 minutes before ischemia and 30 minutes at the beginning of reperfusion recovered significantly LVDP and ±dP/dt as shown in Fig 1B and 1C. The comparison of superimposed LVDP after I/R obtained either from untreated hearts or from hearts treated with Ucn-1, revealed that the peptide decreased the time-to-peak of LVDP, recovered fully its amplitude and accelerated the relaxation comparing to untreated hearts submitted to I/R (Fig 1D and 1E).

**Fig 2:**
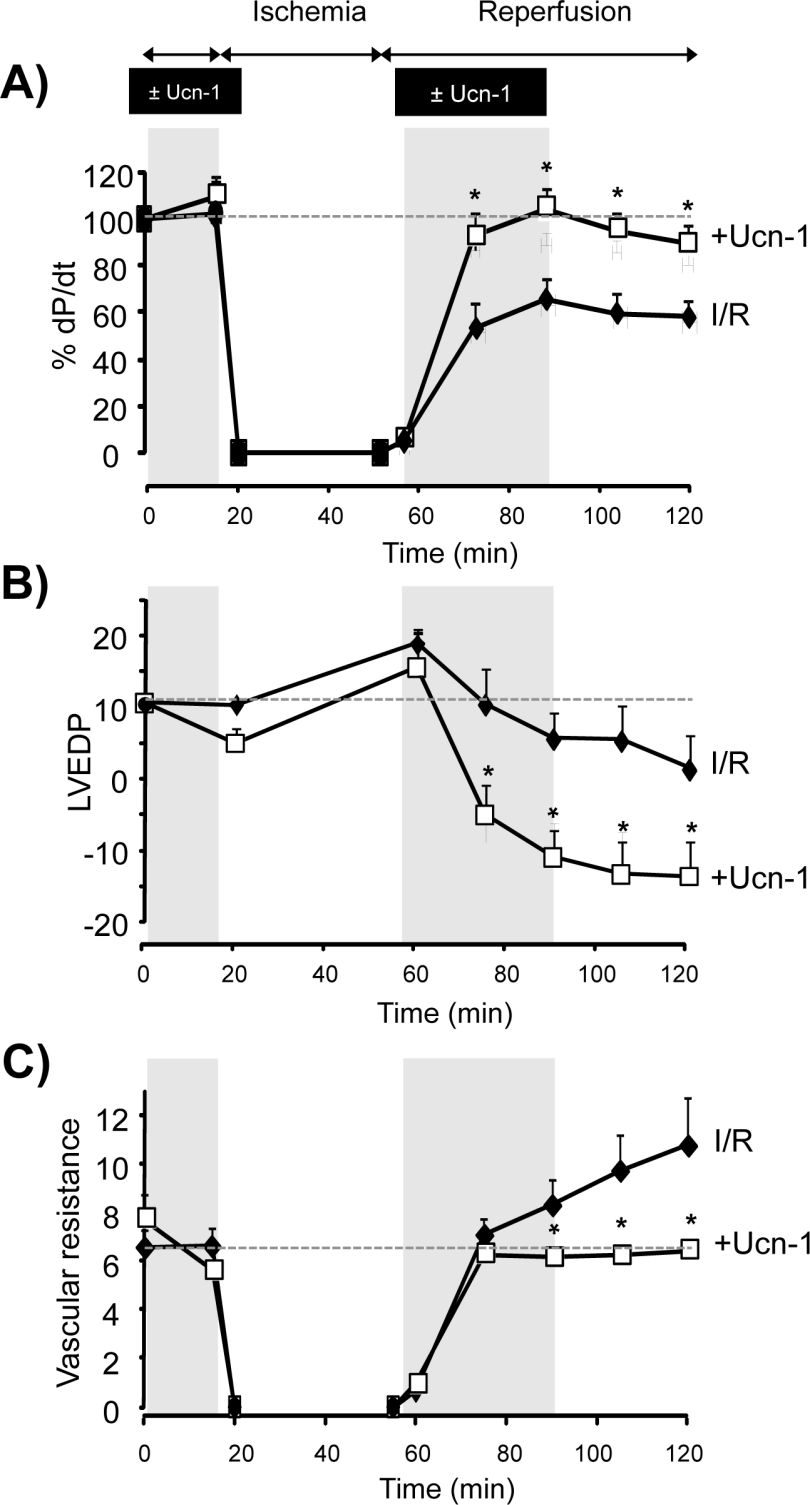
Urocortin-1 improves the hemodynamic function of Langendorff perfused rat hearts submitted to ischemia and reperfusion (I/R). **A**) Graph illustrates the contractility expressed as +dP/dt (%) in hearts submitted to ischemia/reperfusion protocol (I/R, full diamond, n = 9), and in hearts treated with 10 nM Ucn-1 (+Ucn-1, open square, n = 8). Ucn-1 were applied 20 minutes before ischemia and 30 minutes in reperfusion as indicated. **B**) Summary data of the left ventricular end diastolic pressure (LVEDP, mmHg) in the same conditions as in “A”. **C**) Shows the mean of vascular resistance (mmHg*min/ml) changes during the same experiments described in “A”. Dashed lines indicate basal control values for each hemodynamic parameter. Values are means ± s.e.m. “*” indicates significance at p<0.05 of I/R vs +Ucn-1.

Furthermore Fig 2 shows that Ucn-1 administration recovered completely +dP/dt which was sustained in the maximum level in reperfusion (Fig 2A), and improved others hemodynamic parameters such as the left ventricular end diastolic pressure (LVEDP) (Fig 2B), and the coronary vascular resistances whose progressive rise observed in reperfusion was prevented significantly by Ucn-1 (Fig 2C). These cardioprotective effects of Ucn-1 were even maintained 30 minutes after washing out the peptide.

### Urocortin-1 promotes cell survival, regulates apoptosis and necrosis in cardiac myocytes exposed to I/R

**Fig 3:**
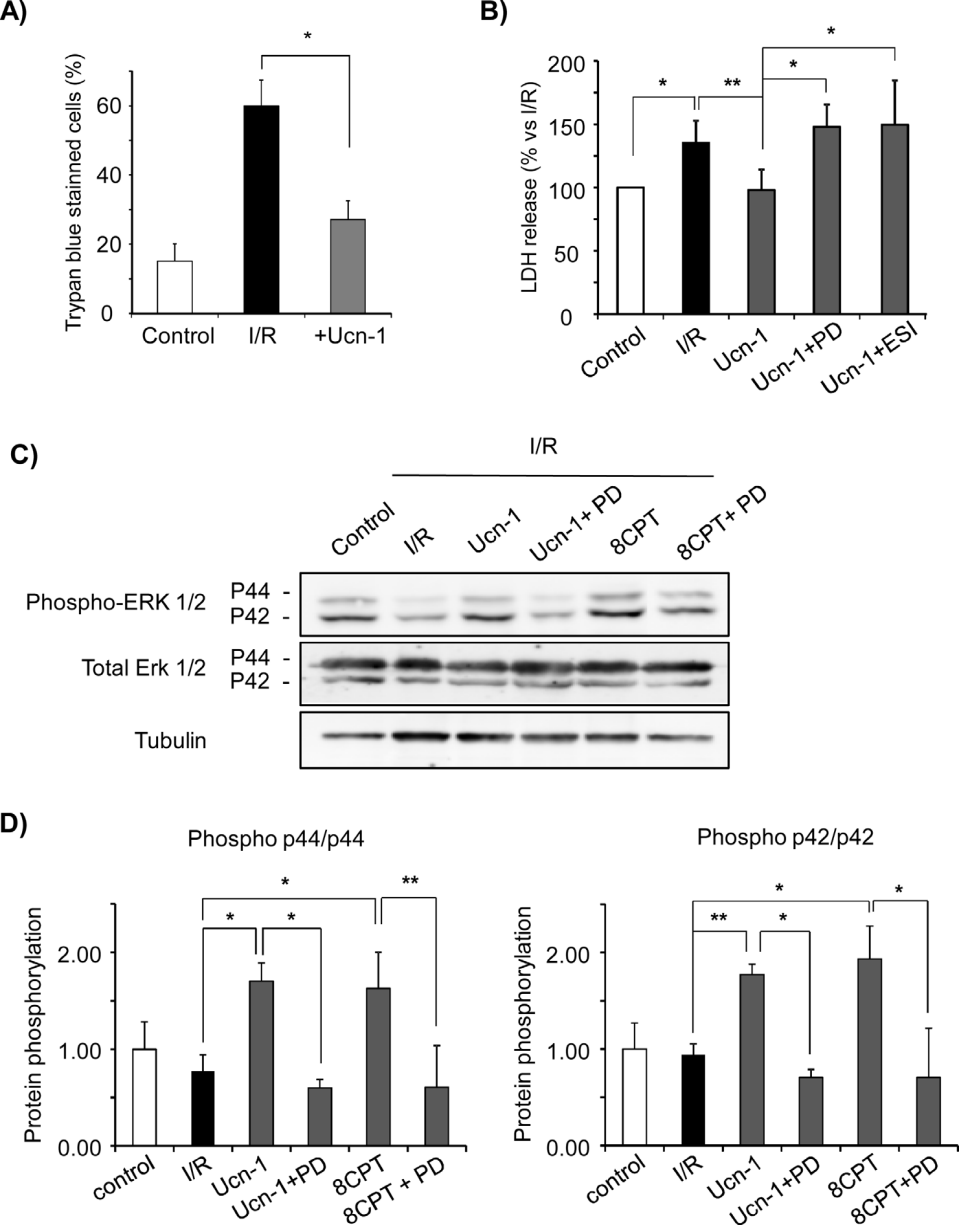
Urocortin-1 increases cell survival, inhibits LDH release by stimulation of ERK1/2 phosphorylation in reperfusion. **A)** Graph summarizes the amount of isolated adult cardiac myocytes stained with trypan Blue. Cells were submitted to I/R (30 minutes/18 hours each) ± Ucn-1 (10 nM). **B)** Bar graph shows the level of LDH release in cardiac myocytes from 5 independent cultures, in control and after I/R (30 minutes/18 hours each) ± Ucn-1 (10 nM). Ucn-1 was also applied in the presence of ERK inhibitor, 5 μM PD 098059 (Ucn-1 + PD), or with Epac2 inhibitor, 5 μM ESI-05 (Ucn-1+ESI). **C)** Western blot and **D)** summary data of ERK 1/2 phosphorylation in control cardiac myocytes and in cells exposed to I/R (30 minutes/18 hours each). Cells were treated with Ucn-1 (10 nM) or with 8CPT (10 μM) alone or in the presence of 5 μM PD 098059 indicated as Ucn-1 + PD, 8CPT + PD respectively. Data are given as means ± s.e.m. from three independent cardiac myocytes cultures. “*” and “**” indicate significance at p<0.05, and p<0.01 respectively.

To further evaluate the cardioprotection exerted by Ucn-1, we examined cardiac myocytes viability using several approaches. First, we estimated trypan blue staining in cardiac myocytes subjected to I/R. Fig 3A shows a significant increase in trypan blue positive staining in cells submitted to I/R as compared to control. However, cells pretreatment with Ucn-1 (10 nM) decreased significantly the number of trypan blue positive cells. Next, to verify the trypan blue finding cell death was also assessed by measurement of LDH release. Cells exposition to I/R resulted in significant increase of LDH levels, meanwhile Ucn-1 (10 nM) significantly reduced the release of LDH during I/R (Fig 3B).

**Fig 4:**
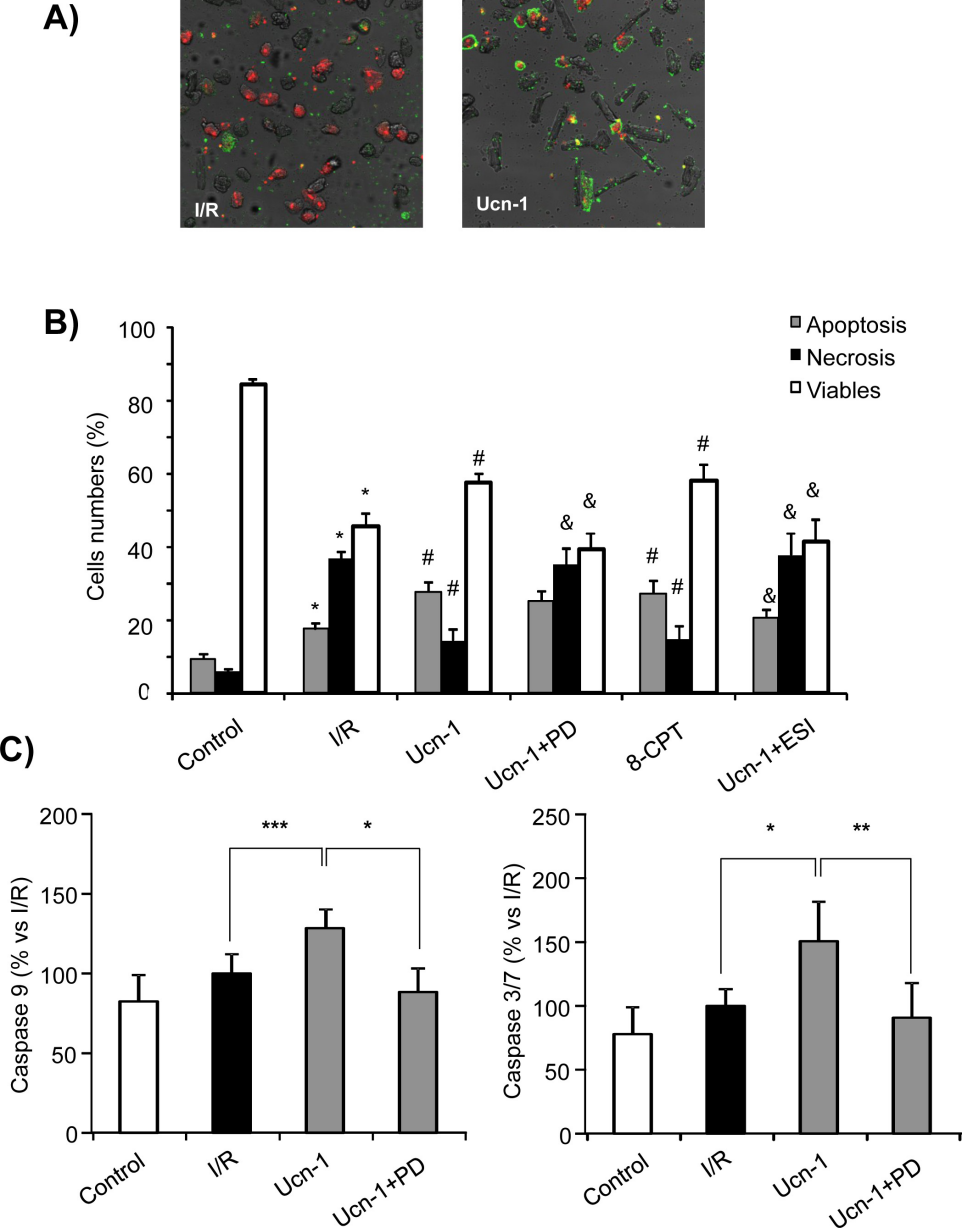
Urocortin-1 effects on cell death and apoptosis. **A)** Representative images of isolated adult cardiac myocytes submitted to I/R (left) and treated with Ucn-1 (right), stained with annexin V-FITC (green) and PI (red). **B)** Shows the summary data from untreated cells (control) and cells exposed to I/R (30 minutes/18 hours each) ± Ucn-1 (10 nM) alone or in presence of 5 μM PD 098059 (Ucn-1 + PD) or 10 μM ESI-05 (Ucn-1 + ESI). 8CPT bars are for cells exposed to I/R (30 minutes/18 hours each) treated with 8CPT (10 μM). White bars indicate viable cells; black bars are for necrosis; and grey bars indicate apoptosis. n = 5 cultures. “*” indicates significance at p<0.05 of I/R vs control. “#” indicates significance at p<0.05 of Ucn-1 and 8CPT comparing to I/R. “&” indicates significance at p<0.05 of Ucn-1 comparing to Ucn-1+PD or to Ucn-1+ESI. **C)** Graphs show caspase 9 and 3/7 activation in similar experiments as in “B. n = 5 cultures. Data are given as means ± s.e.m. “*”, “**” and “***” indicate significance at p<0.05, p<0.01 and p<0.001 respectively.

In addition, we used annexin V-FITC staining to label apoptotic cells in combination with red-fluorescent PI (propidium iodide) that stains late apoptotic/necrotic cells. Representative images and data analysis in Fig 4A and 4B show that cells subjected to I/R resulted in a significant loss of cell viability and a marked increase of the PI positive population, reflecting higher level of cell death by necrosis. Whereas, Ucn-1 (10 nM) treatment of cardiac myocytes exposed to I/R increased the amount of viable cells. Interestingly, Ucn-1 promoted a significant decrease in PI positive cells (necrosis) but it increased early apoptosis as indicated by annexin V positive staining, suggesting that Ucn-1 enhanced cell viability and shifted cell death from necrosis to apoptosis in cells exposed to I/R. Next, we examined caspases activity using an enzymatic-based assay. Fig 4C shows that Ucn-1 enhanced significantly caspase 9 and 3/7 activity in cells exposed to I/R, consistent with annexin-FITC V/PI observations. However, the activation of caspase 8 was not affected by Ucn-1 as shown in S1 Fig.

### Signaling pathway involved in the protection afforded by urocortin-1

We examined the contribution of ERK1/2 pathway in Ucn-1 cardioprotective effects. Fig 3C and 3D shows that the addition Ucn-1 (10 nM) enhanced the phosphorylation of both ERK p42 and p44 isoforms in isolated cardiac myocytes submitted to I/R. Ucn-1 induced phorphorylation of ERK 1/2 was potently reduced by ERK1/2 inhibitor PD 098059 (5 μM) (17). Interestingly, cells pretreatment with PD 098059 prevented Ucn-1 cytoprotective effects as LDH release (Fig 3B), cell viability and necrosis (Fig 4B) and caspase 9 and 3/7 activation (Fig 4C).

Because Epac activates the Ras1-ERK1/2 pathway [18], we also investigated whether Epac signals ERK1/2 activation in Ucn-1 treated cardiac myocytes. Fig 3C and 3D shows that cells incubation with 8CPT (10 μM), the specific agonist of Epac [19], also stimulated ERK1/2 phosphorylation in I/R, which was inhibited significantly by PD 098059. Fig 4B also shows that 8CPT (10 μM) mimicked the effect of Ucn-1 on cell viability, apoptosis and necrosis. To further confirm Epac role in Ucn-1 action we tested ESI-05 (10 μM), considered as the most specific inhibitor of Epac2 [20]. As shown in Figs 3B and 4B, cells pretreatment with ESI-05 abolished Ucn-1 cytoprotective effects on LDH release cell viability, apoptosis and necrosis. The addition of PD 098059 or ESI-05 in cardiac myocytes subjected to I/R, but in the absence of Ucn-1, didn’t change cell viability, apoptosis and necrosis as illustrated in S2 Fig.

### Urocortin-1 alters apoptotic gene expression

**Fig 5:**
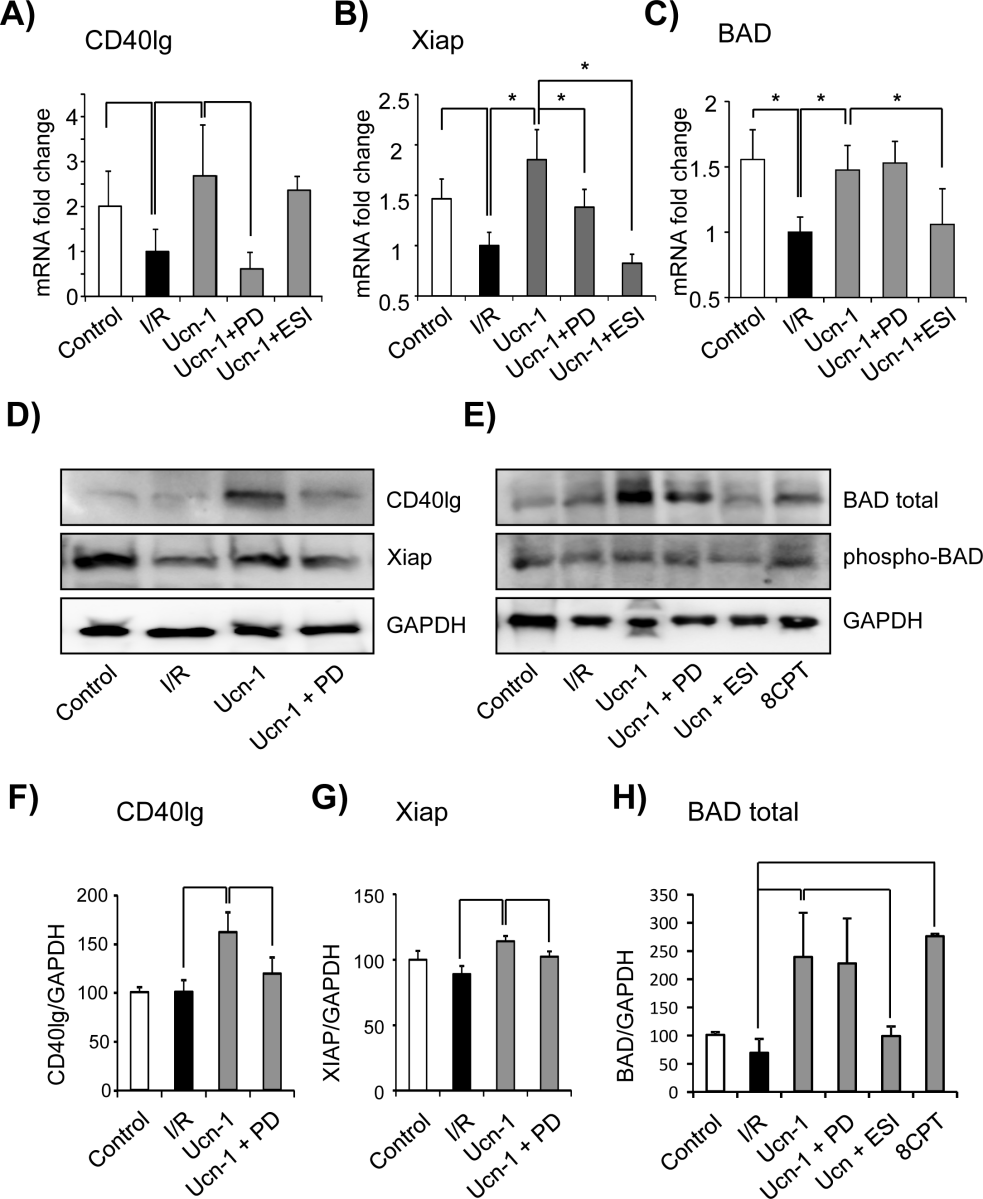
Urocortin-1 effects on the expression of CD40lg, Xiap, and BAD. **A, B and C)** Bar graphs show mRNA levels of CD40lg, Xiap and BAD respectively from 5 cultures of cardiac myocytes in control and after I/R (30 minutes/18 hours each) ± Ucn-1. Ucn-1 (10 nM) was also applied in presence of 5 μM PD 098059 (Ucn-1 + PD) or 5 μM ESI-05 (Ucn-1 + ESI). **D to H)** Representative western blot and summary data of CD40lg, Xiap, and BAD in similar conditions as in A, B and C. 8CPT (10 μM) was applied instead of Ucn-1 to activate Epac. Samples were from 3 different cardiac myocytes isolation. “*”, and “**” indicate significance at p<0.05 and p<0.01 respectively

To get more insight into the mechanism involved in Ucn-1 cytoprotection and apoptosis, we analyzed the expression of 84 key genes involved in apoptosis using a PCR based micro-array technique. S3 Fig shows that the expression of several genes changed in cells undergoing the same protocol of I/R and incubated with Ucn-1 (10 nM) comparing to untreated cells. Next, we verified by RT-PCR analysis the impact of I/R on CD40lg, Xiap and BAD genes expression and their possible modulation by Ucn-1. We observed that mRNA expression of CD40lg (Fig 5A), Xiap (Fig 5B) and BAD (Fig 5C) were significantly decreased in cells exposed to I/R, whereas cells treatment with Ucn-1 recovered completely these genes expression. The effect of Ucn-1 on CD40lg and Xiap were abolished by ERK1/2 inhibition with PD 098059 (5 μM). Meanwhile, Epac2 inhibition with ESI-05 (10 μM) blocked the effect of Ucn-1 on Xiap but not on CD40lg expressions. Furthermore, the evoked upregulation of BAD by Ucn-1 was inhibited in the presence of ESI-05 (10 μM) but not when cells were treated with PD 098059 (5 μM).

Next, the analysis of protein expression showed that CD40lg, Xiap and Bad levels were not significantly affected by I/R, however the administration of Ucn-1 enhanced significantly the expression of CD40lg and Xiap protein in ERK1/2 dependent manner, since treatment with PD 098059 (5 μM) prevented Ucn-1 effects as shown in Fig 5D, 5F and 5G). At the same time, Ucn-1 enhanced potently BAD protein expression comparing to untreated cells exposed to I/R (Fig 5E and 5H), which was inhibited by ESI-05 (10 μM) but not by PD 098059 (5 μM) confirming the implication of Epac2 but not ERK1/2. 8CPT (10 μM) cells treatment also mimicked the effect of Ucn-1 by enhancing BAD expression. Since BAD activity depends on its phosphorylation state, we further examined the amount of serine phosphorylated protein in Ucn-1 treated cardiac myocytes. Fig 5E and summary data in S4 Fig show that Ucn-1 didn’t affect BAD phosphorylation, although the amount of free activated protein was significantly increased in cells treated with Ucn-1 and 8CPT compared to cells submitted only to I/R.

## Discussion

Clinical search for new heart protection strategies and new endogenous cardioprotector is still of major relevance. There is continuous interest in developing novel therapeutic targets that potentially protect the heart against I/R injuries and complications [21, 22]. Several studies have demonstrated that different pre or post- conditioning strategies effectively protect the heart and reduce infarct size after an ischemic insult [23]. Urocortin is recognized as a potential therapeutic agent thanks to its powerful cardioprotective properties, involving different intracellular signaling pathways [24, 25].

Our experimental data from Langendorff-perfused rat hearts showed that Ucn-1, given both before and after a global ischemic insult, was able to recover heart contractility, to preserve vascular resistance, and to improve the ventricular diastolic pressure, indicative of its prevention of hypercontracture and rigor. Ucn-1 effectively sustained rat hearts hemodynamics properties and protected it from I/R injury consistent with the effect of Ucn-2 described in previous studies [24, 25].

The degree of cardiac dysfunction after I/R injury reflects the level of cardiac myocytes lesion related to subsequent cell death, scare formation and a worse long-term outcome. Here, we demonstrated that Ucn-1 exerted significant cytoprotective effects as it efficiently decreased trypan blue cell staining, inhibited LDH release, and enhanced cell viability by the modulation of apoptosis and necrosis. Ucn-1 administration decreased significantly necrotic cells but it increased apoptotic cells number. In fact, while Ucn-1 increased cell viability it shifted cell death from necrosis to apoptosis, a more programmed and controlled cell death. Ucn-1 promotion of apoptosis in detriment of necrosis is supposed more beneficial to reduce the impact of I/R lesions as discussed earlier [26]. Apoptosis is a known form of cell death mediated through sequential activation of the caspase cascade. Consistently, Ucn-1 stimulated caspase 3/7 and caspase 9, but not caspase 8 that is associated to death receptors-mediated apoptosis [27]. These data is contrary to Ucn-2 reduction of caspase 3 levels due to a significant induction of natriuresis and decreased tissue mediators of inflammation [10, 28]. Ucn-1 stimulation of caspase 3/7 and 9 findings was supported with Ucn-1 regulation of apoptotic genes expression. Actually, we observed that cardiac myocytes incubation with Ucn-1 preserved the expression of the well known anti-apoptotic genes CD40lg and Xiap [29, 30], as well as the pro-apoptotic BAD [29], which were down-regulated in cells exposed to I/R. Furthermore, at protein levels Ucn-1 also upregulated Xiap, CD40lg, and BAD. The levels of BAD phosphorylation known to allow its confinement to the cytoplasm and the inhibition of BAD-dependent death [31, 32], was not affected in Ucn-1 treated cells. However, the increase in total amount of BAD protein under Ucn-1 treatment was accompanied also by an increase in the amount of dephosphorylated (free) BAD protein, which might explain the observed apoptosis annexin/PI and caspase experiments. Similarly, the activation of BAD by Ucn-1 and Ucn-2 was previously described in macrophage [33] but not yet in heart. On the other hand, Ucn-1 upregulation of anti-apoptotic genes CD40lg and Xiap explains its beneficial effects on cell survival and necrosis. To our knowledge, this study is the first to involve CD40lg and Xiap in Ucn-1 cardioprotective effects. In this study, we noticed that the changes in mRNA levels of apoptotic genes during cells exposition to in I/R were more evident than at the protein levels, which could be due to the complex mechanism of post-transcriptional mechanisms involved in turning mRNA into protein [34].

Moreover, we demonstrated that Ucn-1 implicated Epac2 and ERK1/2 in its cardioprotective actions. ERK1/2 role as a survival pathway in cardiac cells is widely accepted [10–12], nevertheless only few reports directly investigated its activation during reperfusion [35, 36]. In this study, we demonstrated that under I/R, Ucn-1 activated significantly ERK1/2, whose inhibition by PD 098059 dramatically prevented Ucn-1 protective effects in Langendorff perfused hearts (data not shown), and abolished Ucn-1 cytoprotection action supporting the importance of ERK1/2 in mediating urocortin effects, in accordance with others studies [12, 13]. Indeed, we demonstrated that ERK1/2 are also implicated in Ucn-1 inhibition of LDH release, cell viability, caspase activation, and in the modulation of CD40lg and Xiap expression. Furthermore, very few studies have focused on the role of Epac in urocortin signaling pathway [9, 37, 38]. However, the role of Epac in cardioprotection is still under debate. Recently, a cardioprotective role of β-adrenergic signaling via cAMP/Epac/Rap1/Rac/ERK pathway has been described in mice [39]. Epac activation was also associated with cardiac myocytes protection from nitric oxide-induced apoptosis [39, 40]. Here, we provided different data indicating Epac2 involvement in Ucn-1 cardioprotective effects by the use of ESI-05 as a selective inhibitor for Epac2 isoform (for review see [20]). We found that ESI-05 inhibited the effects of Ucn-1 on LDH release and prevented Ucn-1 restoration of Xiap and BAD expression. Our data suggest for the first time an important role for Epac2 in Ucn-1 heart improvement from I/R lesions, which is consistent with the increasing numbers of studies that propose an important role of Epac in heart [6–8, 41]. Finally, Epac as a GTP exchange factor is known to activate Rap1-GTPase by catalyzing the formation of Rap1-GTP and further stimulation of ERK. Herein, we demonstrated that Epac activation with 8CPT mimicked the effect of Ucn-1 on ERK1/2 activation in I/R, which suggested that Epac is upstream of ERK1/2 signaling pathway.

In conclusion, we demonstrated that the administration of Ucn-1 before ischemia and during the first minutes of re-oxygenation produced significant long-lasting protective effects, mediated by a complex signaling pathway that involve Epac2 and ERK1/2. Ucn-1 promotion of cell survival and apoptosis might be beneficial since apoptosis is considered a highly regulated energy-consuming process, required for controlled and programmed cell death, which will limit cardiac cell loss and posterior inflammation processes in I/R. Together with the other cardioprotective actions of urocortin including vasorelaxation of human coronary artery [42], the positive inotropism [9], and the decrease in renin activity, aldosterone, vasopressin, endothelin1 and atrial and B-type natriuretic peptides [43], all hallmark events shown to diminish the destroying effect of I/R injury, certainly confirm urocortin as a valuable target for the treatment of diseases associated with cardiac dysfunction under post-ischemic insults.

## Supporting Information

S1 Fig Urocortin-1 doesn’t activate caspase 8. Graph shows caspase 8 activity analyzed from cells exposed to ischemia and reperfusion (I/R; 30 min/18 h each) ± urocortin-1 (Ucn-1, 10 nM). Ucn-1 (10 nM) was also applied in presence of 5 μM PD 098059 (Ucn-1+PD). Data are given as means ± s.e.m. n = 5 cultures. “ns” indicates that values are not significantly different from control.
(TIF)

S2 Fig Effects of PD 098059 and ESI-05 on cell viability, apoptosis and necrosis. Graph shows summary data from untreated cells (control), cells exposed to I/R (30 minutes/18 hours), cells exposed to I/R in presence of 5 μM PD 098059 (I/R+ PD) or 10 μM ESI-05 (I/R + ESI). “*” indicates significance between control and different treatment at p<0.05. “ns” indicates that values are not statistically different between I/R and I/R+ESI or I/R +PD. Values are means ± s.e.m. n = 4 cultures.
(TIF)

S3 Fig Fold change of the expression of 84 key genes involved in apoptosis. PCR-based array was performed in samples from cardiomyocytes exposed to I/R and treated or not with Ucn-1 (10 nM) as described in cell treatment protocols in material and methods.
(TIF)

S4 Fig Urocortin-1 effects BAD phosphorylation. A) Bar graph shows the analysis of phospho-BAD level from western blot experiments.Protein samples were from cells exposed to ischemia and reperfusión (I/R; 30 min/18 h each) ± urocortin-1 (Ucn-1, 10 nM). Ucn-1 (10 nM) was also applied in presence of 5 μM PD 098059 (Ucn-1+PD) and 5 μM ESI-05 (Ucn-1+ESI). 8CPT (10 μM) was applied instead of Ucn-1 to activate Epac B) Analysis of total BAD and non phosphorylated free BAD in the same experiments as in “A”. Values are means ± s.e.m. from 3 different experiments.
(TIF)

## Abbreviations

cAMP: cyclic AMP
BAD: Bcl-2-associated death promoter
CD40lg: CD40 ligand
CRF: corticotropin-releasing factor
CRF-R1,2: corticotropin releasing factor receptor 1,2
dP/dt: derivative of left ventricular pressure
Epac: exchange protein directly activated by cAMP
ERK1/2: extracellular signal–regulated kinases ½
GEF: guanine nucleotide exchange factor
I/R: ischemia/ reperfusion
LDH: lactate dehydrogenase
LVDP: left ventricular developed pressure
LVEDP: left ventricular end diastolic pressure
MAPK: mitogen activated protein kinase
PKA: protein kinase A
PKCε: protein Kinase C epsilon
Ucn-1: urocortin-1
Xiap: X-linked inhibitor of apoptosis protein
