# Supplementary figures and images for: Urocortin-1 Mediated Cardioprotection Involves XIAP and CD40-Ligand Recovery: Role of EPAC2 and ERK1/2
Source: PLoS One. 2016 Feb 3;11(2):e0147375. doi: 10.1371/journal.pone.0147375 (PMC4739601; doi:10.1371/journal.pone.0147375)

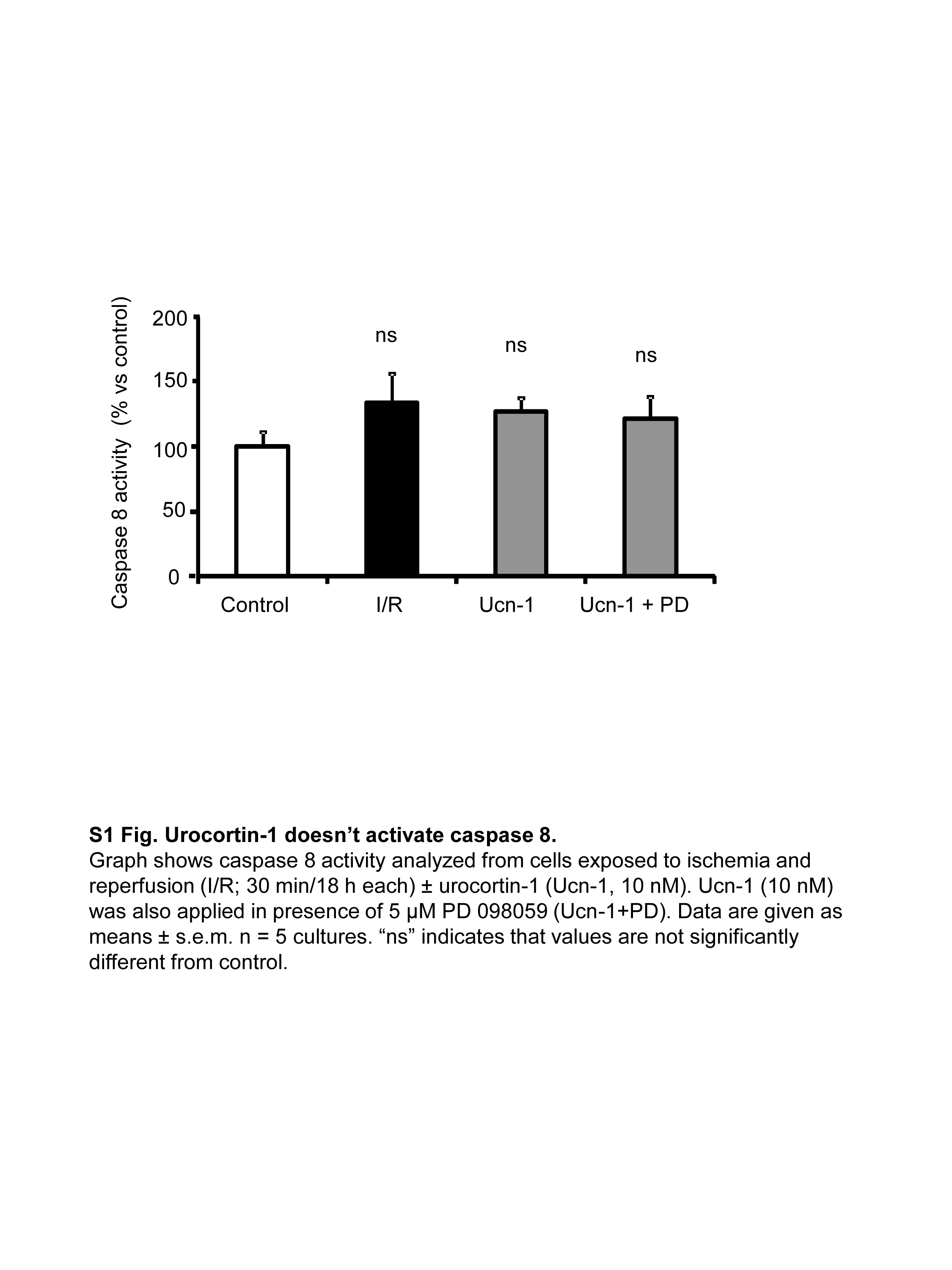

Supplement: S1 Fig — Graph shows caspase 8 activity analyzed from cells exposed to ischemia and reperfusion (I/R; 30 min/18 h each) ± urocortin-1 (Ucn-1, 10 nM). Ucn-1 (10 nM) was also applied in presence of 5 μM PD 098059 (Ucn-1+PD). Data are given as means ± s.e.m. n = 5 cultures. “ns” indicates that values are not significantly different from control. (TIF) [file pone.0147375.s001.tif]

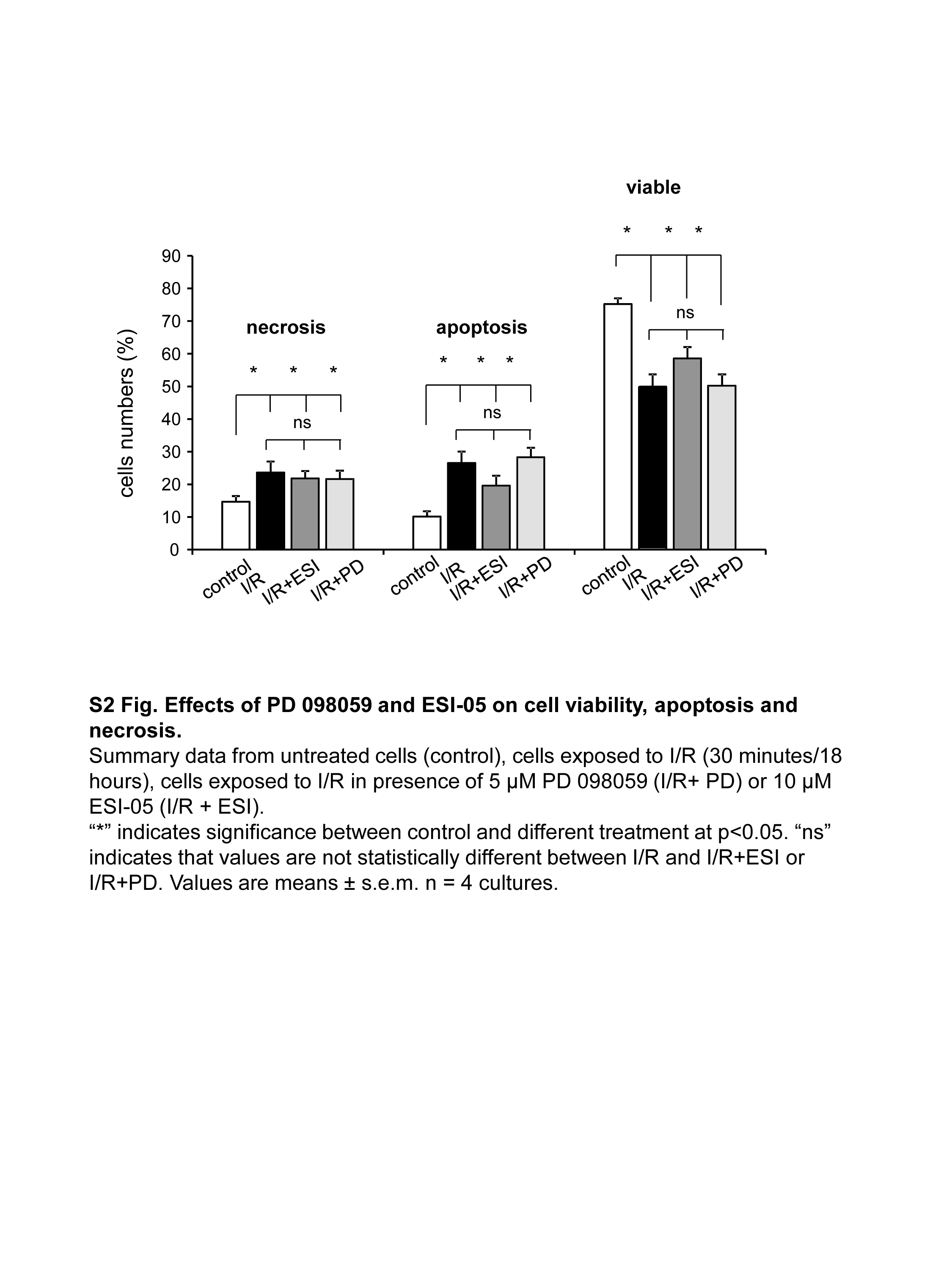

Supplement: S2 Fig — Graph shows summary data from untreated cells (control), cells exposed to I/R (30 minutes/18 hours), cells exposed to I/R in presence of 5 μM PD 098059 (I/R+ PD) or 10 μM ESI-05 (I/R + ESI). “*” indicates significance between control and different treatment at p<0.05. “ns” indicates that values are not statistically different between I/R and I/R+ESI or I/R +PD. Values are means ± s.e.m. n = 4 cultures. (TIF) [file pone.0147375.s002.tif]

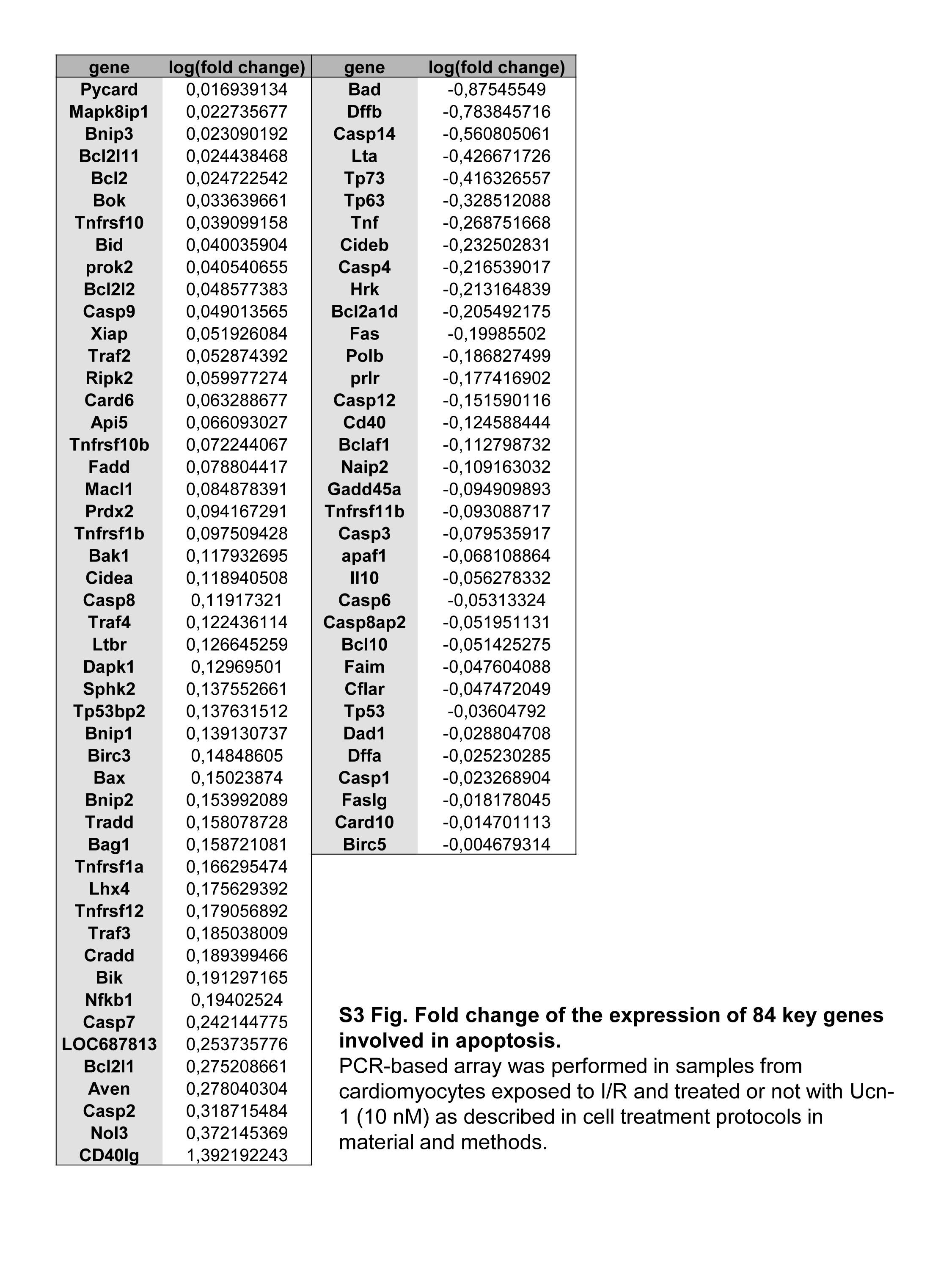

Supplement: S3 Fig — PCR-based array was performed in samples from cardiomyocytes exposed to I/R and treated or not with Ucn-1 (10 nM) as described in cell treatment protocols in material and methods. (TIF) [file pone.0147375.s003.tif]

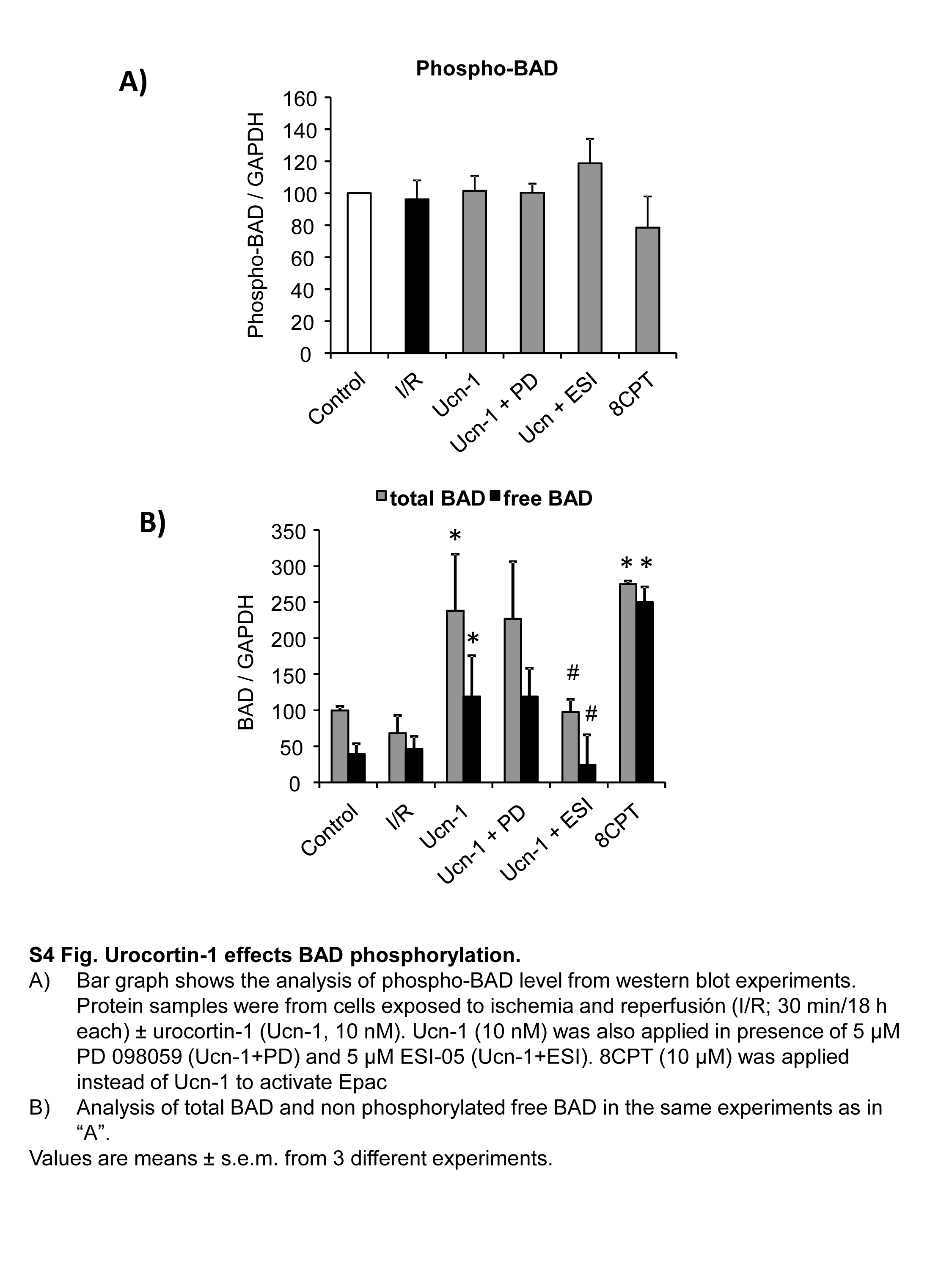

Supplement: S4 Fig — A) Bar graph shows the analysis of phospho-BAD level from western blot experiments.Protein samples were from cells exposed to ischemia and reperfusión (I/R; 30 min/18 h each) ± urocortin-1 (Ucn-1, 10 nM). Ucn-1 (10 nM) was also applied in presence of 5 μM PD 098059 (Ucn-1+PD) and 5 μM ESI-05 (Ucn-1+ESI). 8CPT (10 μM) was applied instead of Ucn-1 to activate Epac B) Analysis of total BAD and non phosphorylated free BAD in the same experiments as in “A”. Values are means ± s.e.m. from 3 different experiments. (TIF) [file pone.0147375.s004.tif]
